# Sensing telomerase: From *in vitro* detection to *in vivo* imaging

**DOI:** 10.1039/c6sc04801c

**Published:** 2016-12-13

**Authors:** Li-juan Wang, Fei Ma, Bo Tang, Chun-yang Zhang

**Affiliations:** a College of Chemistry , Chemical Engineering and Materials Science , Collaborative Innovation Center of Functionalized Probes for Chemical Imaging in Universities of Shandong , Key Laboratory of Molecular and Nano Probes , Ministry of Education , Shandong Provincial Key Laboratory of Clean Production of Fine Chemicals , Shandong Normal University , Jinan 250014 , China . Email: cyzhang@sdnu.edu.cn ; Email: tangb@sdnu.edu.cn ; Fax: +86 0531 82615258 ; Tel: +86 0531 86186033

## Abstract

Telomerase is a ribonucleoprotein reverse transcriptase that is responsible for maintaining the telomere length in cells. Telomerase overexpresses in almost all malignant tumor cells, and it has become a promising biomarker and a potential therapy target for cancers. Consequently, accurate and efficient quantification of the telomerase is highly essential to medical diagnostics and therapeutics. Recently, a series of novel telomerase detection methods with excellent performance have been developed, but a overview of *in vivo* telomerase detection methods is lacking. In this Minireview, we summarize the emerging strategies for telomerase assays in the last five years, including both *in vitro* assays and *in vivo* imaging methods, and discuss their future directions as well.

## Abstract 2

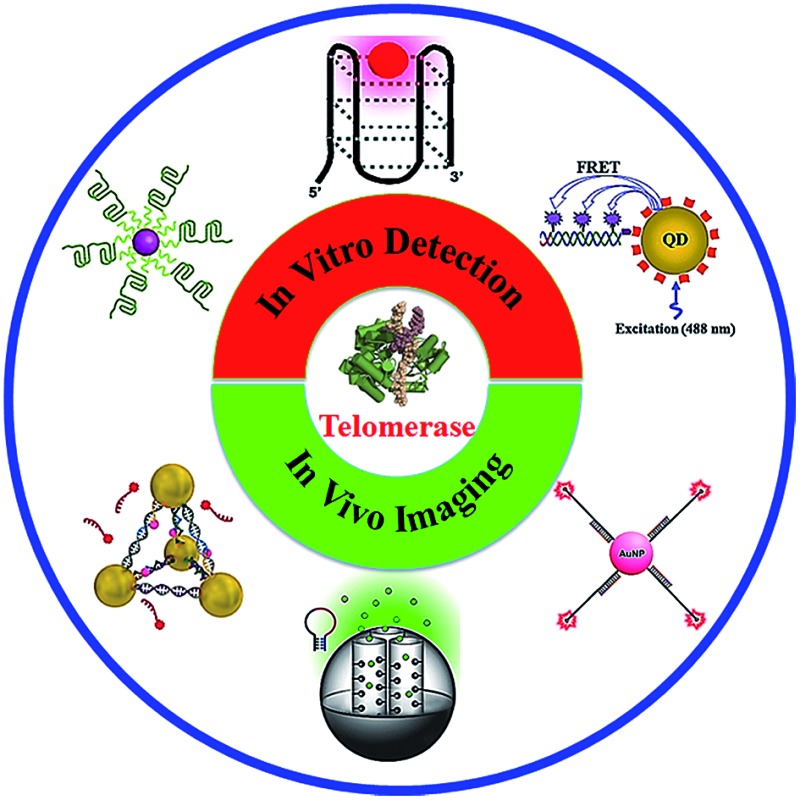
We reviewed recent advances in telomerase assays including both *in vitro* detection and *in vivo* imaging.

## 1 Introduction

At the end of a chromosome, there is a region of repetitive nucleotide sequences (TTAGGG for human cells) called the telomere.^1^ During natural cell division, the telomere length may be progressively shortened due to chromosome replication, leading to cellular aging and senescence.^2^ This shortened telomere may be replenished by telomerase.^3^ Telomerase is a ribonucleoprotein reverse transcriptase which consists of two molecules: the catalytic subunit of telomerase reverse transcriptase (TERT) and the TERT template of telomerase RNA (TERC).^4,5^ TERT employs TERC to add the repeating sequence to the 3′ end of a chromosome, preventing the shortening of the natural telomere.^6^ Notably, the Nobel Prize in Physiology/Medicine (2009) was awarded to Elizabeth H. Blackburn, Carol W. Greider and Jack W. Szostak for their discovery of how chromosomes are protected by telomeres and the enzyme telomerase.^7–9^ Recent research has demonstrated that the activation of telomerase activity may cause cellular immortality and cancers,^10^ while the inactivation of the telomerase may accelerate cell aging.^11^ The telomerase activity is repressed in most somatic human cells, while an elevated telomerase level is observed in over 85% of cancer cells,^12^ including gastric cancer,^13^ colorectal cancer,^14^ cervical cancer,^15^ pancreatic cancer,^16^ breast cancer,^17^ urothelial cancer,^18,19^ and high-risk neuroblastoma.^20^ Therefore, the telomerase may function as a universal biomarker for cancer diagnosis. Weinberg and colleagues found that the inhibition of telomerase by a mutant catalytic subunit of human telomerase may lead to the death of tumor cells,^21^ and more evidence^22^ indicates that telomerase may serve as an attractive target for anti-cancer therapies and anti-telomerase drug development.^23–25^ For example, a telomerase template antagonist, GRN163, can inhibit telomerase activity and cause the suppression of tumor growth.^26^ As a result, the accurate quantification of the telomerase is of great importance to clinic diagnosis and cancer therapy.

Recently, great efforts have been put into the development of efficient methods for telomerase assay since its discovery in 1985.^27^ The most classical method is the polymerase chain reaction (PCR)-based telomeric repeat amplification protocols (TRAPs),^28^ in which telomerase products are amplified and measured by PCR. The TRAP methods, however, are susceptible to the cell extract-induced inhibition. Alternatively, a series of new telomerase assays have emerged in recent years with the progress in bioanalytical chemistry and material sciences. Since the publication of the only review about the telomerase assay in 2012,^29^ great advance has been made in this area, especially in the *in vivo* telomerase assay using novel nanomaterials. In this Minireview, we focus on the development of novel telomerase assays in the last five years (2012–2016). These assays may be divided into two categories: *in vitro* telomerase quantification; and *in vivo* telomerase imaging. We introduce the assay principle and compare their performance. Meanwhile, we discuss the current challenges and future directions as well.

## 2 *In vitro* telomerase assays

In the *in vitro* telomerase assays, the target enzyme is extracted from the cells, and then quantified by various detection approaches including fluorescent, colorimetric, electrochemiluminescent, Raman scattering spectroscopy, and chemiluminescent methods.

### 2.1 Fluorescent assay

The fluorescent method is a widely used strategy for telomerase assay. The telomerase products may be directly/indirectly monitored by fluorescent labels, such as zinc(ii)-protoporphyrin IX (ZnPPIX), SYBR Green (SG), molecular beacon, quantum dot (QD) and Cy5. The fluorescent methods may be divided into three categories: (1) amplification-free assay; (2) amplification-assisted assay; and (3) single QD-based assay.

#### 2.1.1 Amplification-free fluorescent assay

**Fig. 1:**
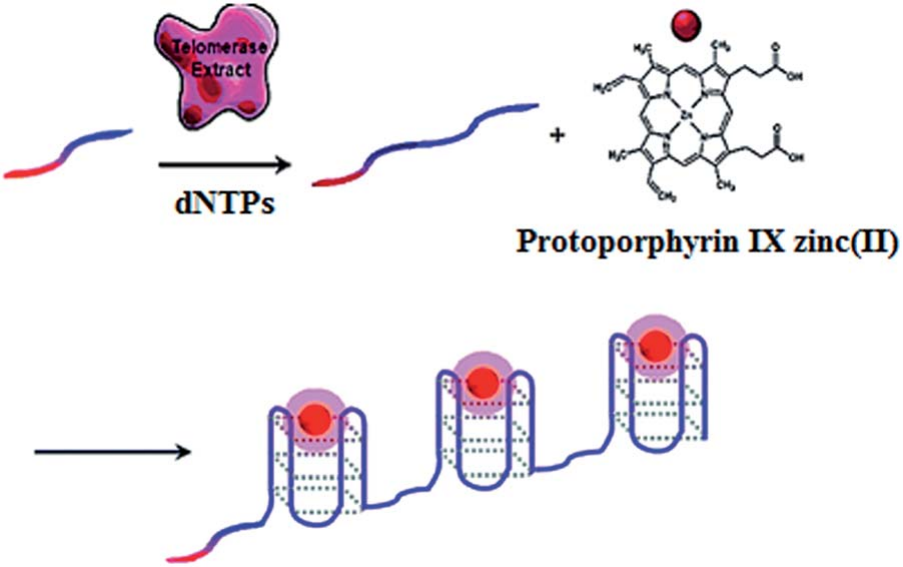
Amplification-free fluorescent detection of telomerase activity using ZnPPIX as the fluorescent agent. Adapted with permission from ref. 30, Copyright 2012 American Chemical Society.

The telomerase is able to generate short tandem repeats of TTAGGG, which can form G-quadruplex oligomers. The integration of ZnPPIX into G-quadruplex may induce the increase of ZnPPIX fluorescence by 9-fold.^30^ Willner demonstrated a label-free fluorescent method for telomerase assay using ZnPPIX (Fig. 1).^30^ In the presence of dNTPs, telomerase extends the primer, producing multiple G-quadruplex sequences which subsequently bind ZnPPIX to generate an enhanced fluorescent signal. This assay is very simple without the involvement of any amplification steps, and it may measure telomerase activity originating from 380 ± 20 cells per μL. In addition, this method is versatile and may be applied for the detection of DNA and adenosine-5′-triphosphate (ATP) using a specific hairpin probe and aptamer.

#### 2.1.2 Amplification-assisted fluorescent assay

**Fig. 2:**
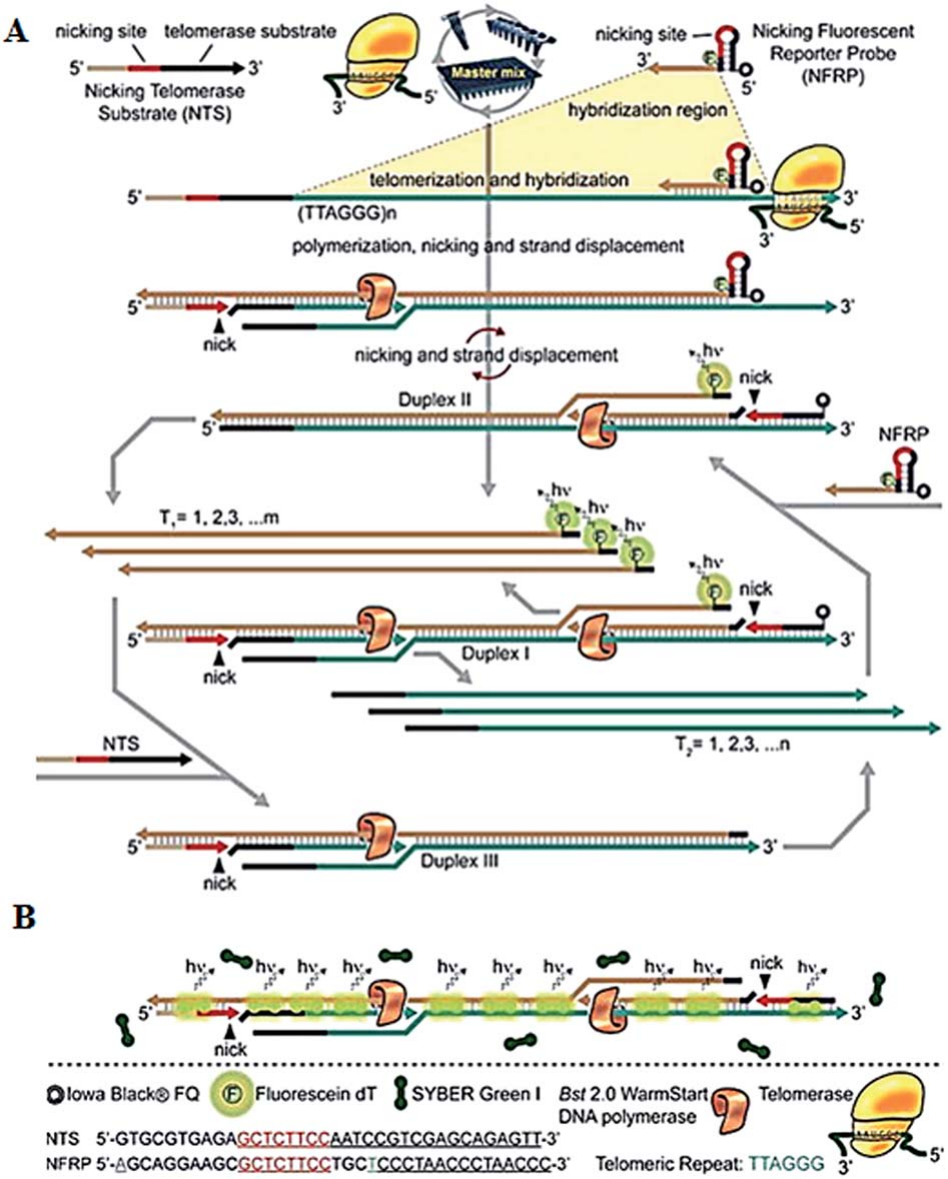
Exponential isothermal amplification-based fluorescent detection of telomerase activity using (A) the NFRP and (B) SYBR Green I dye as the fluorescent probes. Adapted with permission from ref. 31, Copyright 2013 American Chemical Society.

To improve the detection sensitivity, amplification approaches are introduced into the telomerase assays. Weizmann demonstrated the use of exponential isothermal amplification of the telomere repeat (EXPIATR) for real-time fluorescent detection of telomerase activity (Fig. 2).^31^ Unlike PCR amplification in TRAP-based telomerase,^28^ the isothermal amplification does not need precise temperature control and sophisticated instruments.^32^ This assay involves two primers (*i.e.*, the nicking telomerase substrate primer and the nicking fluorescent reporter probe primer) and three enzymes (*i.e.*, DNA polymerase, nicking endonuclease and telomerase), with two primers containing the recognition sequence of the nicking endonuclease, respectively. In the presence of telomerase, the nicking telomerase substrate (NTS) may generate extension products which may function as the template for DNA amplification, initiating an exponential isothermal amplification reaction in the presence of DNA polymerase, nicking endonuclease and two primers. As a result, a number of double-stranded DNA (dsDNA) products are obtained. Telomerase activity can be real-time detected by the nicking fluorescent reporter probe (NFRP) which can monitor the amplification process (Fig. 2A) and SYBR Green I which can stain the amplification products of dsDNAs specifically (Fig. 2B). This assay is very simple, without the involvement of thermal cycling, and it can detect telomerase activity in a single HeLa cell within 25 min.

Zhang demonstrated the use of telomerase-triggered isothermal exponential amplification for highly sensitive detection of telomerase activity.^33^ In this assay, the extension products of the telomerase are amplified by an exponential amplification reaction, generating multiple 8–17 DNAzyme sequences which may be detected by molecular beacons in the presence of cofactor Pb^2+^.^34^ This assay can measure telomerase activity at the single-cell level, and may be further applied for the screening of telomerase inhibitors.

#### 2.1.3 Single QD-based assay

**Fig. 3:**
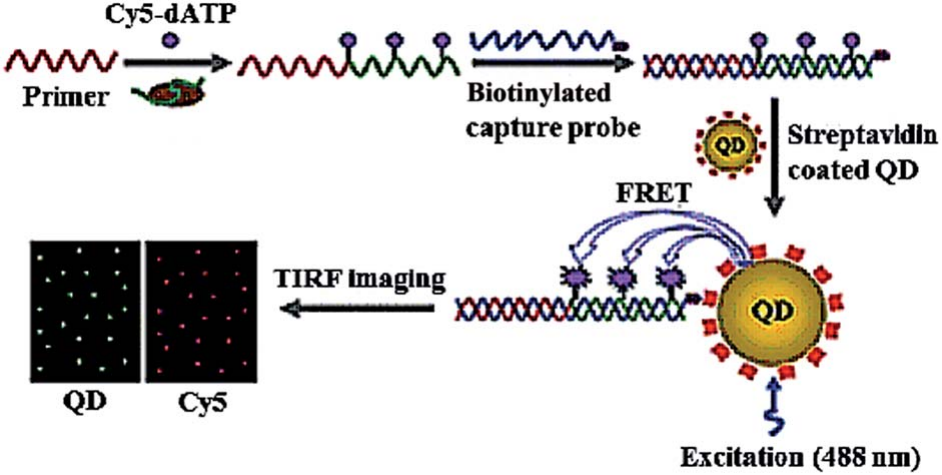
Single QD-based telomerase assay. Adapted with permission from ref. 37, Copyright 2015 The Royal Society of Chemistry.

Due to their high quantum yield, good stability against photobleaching, narrow luminescence bands and size-tunable luminescence spectra, semiconductor quantum dots (QDs) have been widely used for the detection of various biomolecules.^35^ Especially, the integration of fluorescence resonance energy transfer (FRET) with QDs enables the homogeneous detection of DNA, microRNA and proteins.^36^ Zhang developed a single QD-based biosensor for the sensitive detection of telomerase activity (Fig. 3).^37^ In the presence of telomerase, the primer is extended by telomerase and simultaneously labelled by Cy5 with Cy5-dATP as the fuel. The Cy5-labeled extension products hybridize with the biotinylated capture probes and subsequently assemble on the surface of QD *via* biotin–streptavidin interaction to form the Cy5-dsDNA-QD assembly, leading to efficient FRET from the QD donor to the Cy5 acceptor. The telomerase activity may be simply quantified by counting the Cy5 signals at the single-molecule level. This assay is very sensitive, with a detection limit of 7 cells per μL, which is improved by 9-fold compared to the ensemble fluorescence spectrum measurement. This assay can be further applied for the screening of anti-cancer drugs.

### 2.2 Colorimetric assay

**Fig. 4:**
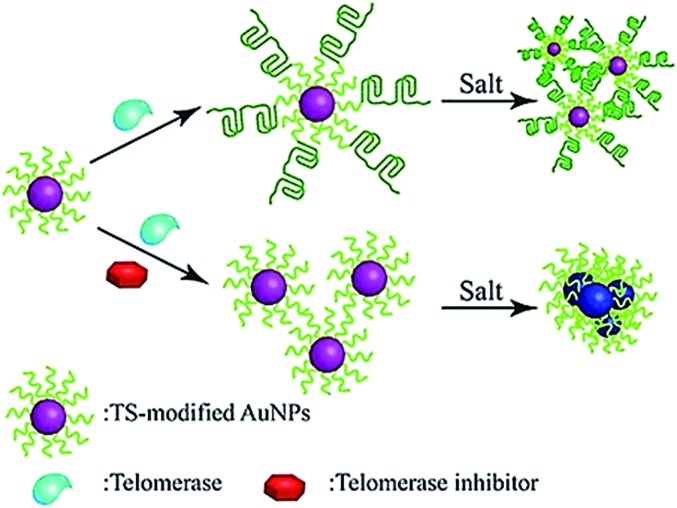
Colorimetric detection of telomerase activity using telomerase primer-modified AuNPs. Adapted with permission from ref. 38, Copyright 2012 Wiley-VCH Verlag GmbH & Co. KGaA, Weinheim.

The colorimetric method possesses the advantages of simplicity and being cost-effective, and its signal can be directly monitored by the naked eye. Qu demonstrated the use of primer-modified gold nanoparticles (AuNPs) for visualization detection of telomerase activity (Fig. 4).^38^ In the absence of telomerase, the primer-modified AuNPs aggregate in a defined salt concentration, leading to the change in color. Meanwhile, in the presence of telomerase, the extension of the primer by telomerase prevents the AuNPs from aggregation, and no color change is observed. This assay enables a simple and fast quantification of telomerase with a detection limit as low as 1 HeLa cell per μL. It can be further used to screen telomerase inhibitors for the discovery of anticancer agents.

In addition, based on the idea that l-cysteine may stimulate the aggregation of AuNPs, Willner developed an AuNP-based colorimetric method for telomerase detection.^39^ In this assay, the enzyme products of telomerase may fold into G-quadruplex structures in the presence of K^+^ ions and hemin, which possesses horseradish peroxidase mimicking functions and may catalyze the oxidation of l-cysteine into cysteine. As a result, the aggregation of AuNPs is prevented, and a distinct color change is observed. The assay can sensitively detect telomerase from 293T cancer cell extracts with a detection limit of 27 cells per μL, providing a potential point-of-care sensing platform for cancer diagnosis.

### 2.3 Electrochemical assay

**Fig. 5:**
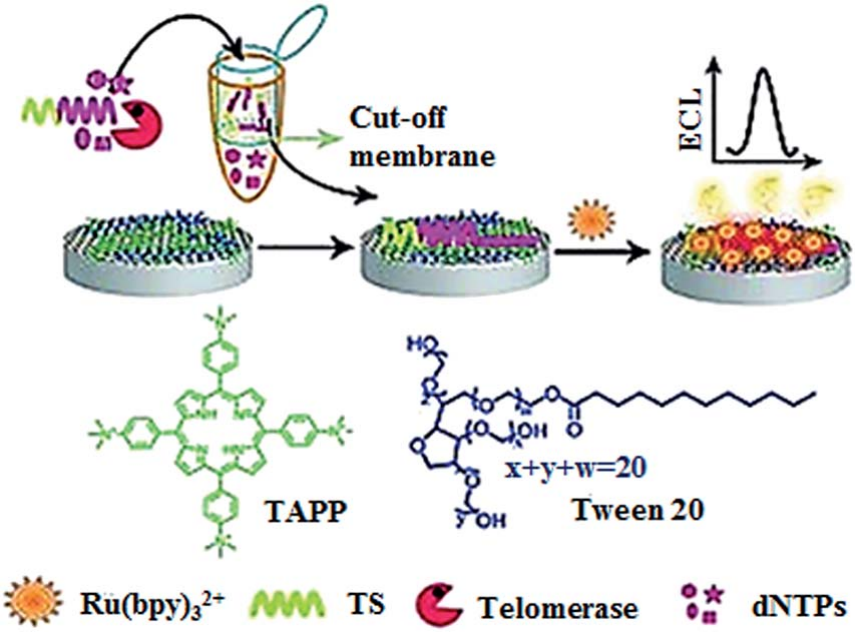
Label-free electrochemiluminenscent detection of telomerase activity using porphyrin-functionalized graphene. Adapted with permission from ref. 41, Copyright 2012 WILEY-VCH Verlag GmbH & Co. KGaA, Weinheim.

The electrochemiluminescent method enables label-free and sensitive detection of biomolecules with a low background signal.^40^ Qu demonstrated the development of an electrochemiluminescent biosensor for the sensitive detection of telomerase activity (Fig. 5).^41^ They introduced the *meso*-tetra-(4-*N*,*N*,*N*-trimethylanilinium) porphyrin (TAPP) which had positively charged groups to prevent the aggregation of graphene. Meanwhile, they employed Ru(bpy)_3_^2+^ as the signal reporter and Tween 20 as the blocking agent to prevent the nonspecific binding of proteins in cell extract. The glassy carbon electrode was modified by TAPP-functionalized chemically converted graphemes (CCG). The negatively charged phosphate backbone and nucleotide base in the telomerase primer made it absorb on the grapheme surface through electrostatic attraction and π–π stacking. Subsequently, the negatively charged DNA brings the positively charged Ru(bpy)_3_^2+^ to the surface of the glassy carbon electrode through electrostatic attraction, generating an ECL signal. The elongation of the primer by telomerase produces a longer oligonucleotide which attracts more Ru(bpy)_3_^2+^ to the electrode surface, resulting in an enhanced ECL signal. This assay can sensitively measure telomerase activity with a detection limit of as low as 10 HeLa cells per mL. Recently, a series of electrochemical methods have been reported for telomerase assay using Methylene Blue^42^ and hexaammineruthenium(iii) chloride^43^ as the reporters. In addition, metal–organic frameworks have been used in the development of electrochemical methods for telomerase assays.^44,45^

### 2.4 Raman scattering spectroscopy-based assay

**Fig. 6:**
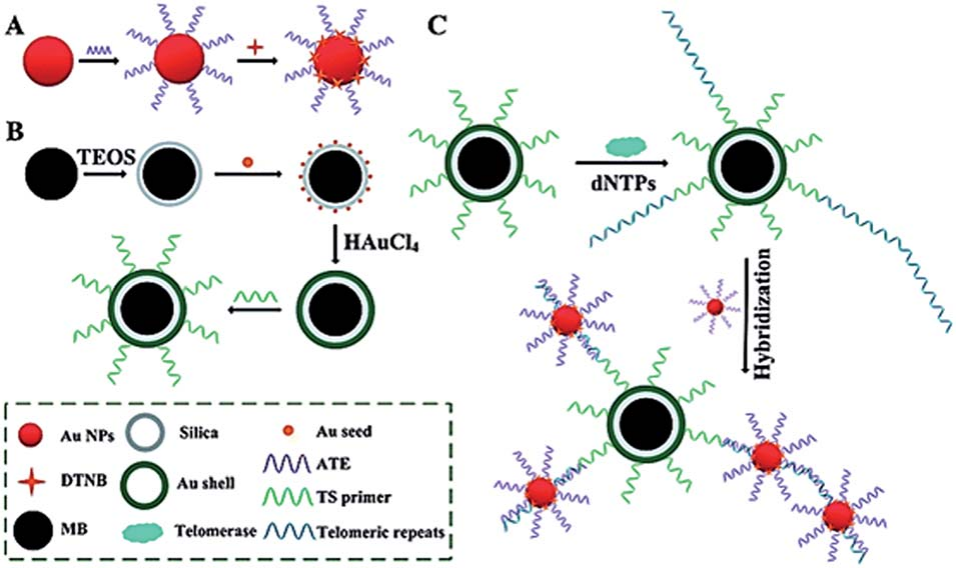
SERS detection of telomerase activity using functionalized AuNPs and magnetic nanobeads. Adapted with permission from ref. 47, Copyright 2014 The Royal Society of Chemistry.

Surface-enhanced Raman scattering (SERS) may provide spectral fingerprint signatures of a specific target without interference from non-specific molecules.^46^ Zong demonstrated the use of SERS for sensitive detection of telomerase activity (Fig. 6).^47^ They prepared reporting gold nanoparticles (AuNPs) and capturing gold (Au) shell-coated magnetic nanobeads (MBs), respectively. The reporting AuNPs are modified with the Raman reporter 5,5′-dithiobis (2-nitrobenzoic acid) (DTNB) and a telomeric repeat complementary oligonucleotide (ATE); the capturing Au shell-coated MBs are modified with a telomerase-substrate oligonucleotide (TS primer). In the presence of telomerase, the TS primers are elongated, generating tandem telomeric repeats. The hybridization of telomeric repeats with ATEs leads to the formation of AuNP–DTNB–MB complexes. After magnetic separation, a distinct SERS signal can be detected. Meanwhile, in the absence of telomerase, no telomeric repeat is produced, and no SERS signal is observed due to the absence of the AuNP–DTNB–MB complex. This assay is very sensitive and even the telomerase from 1 tumor cell per mL can be detected.

### 2.5 Chemiluminescent assay

**Fig. 7:**
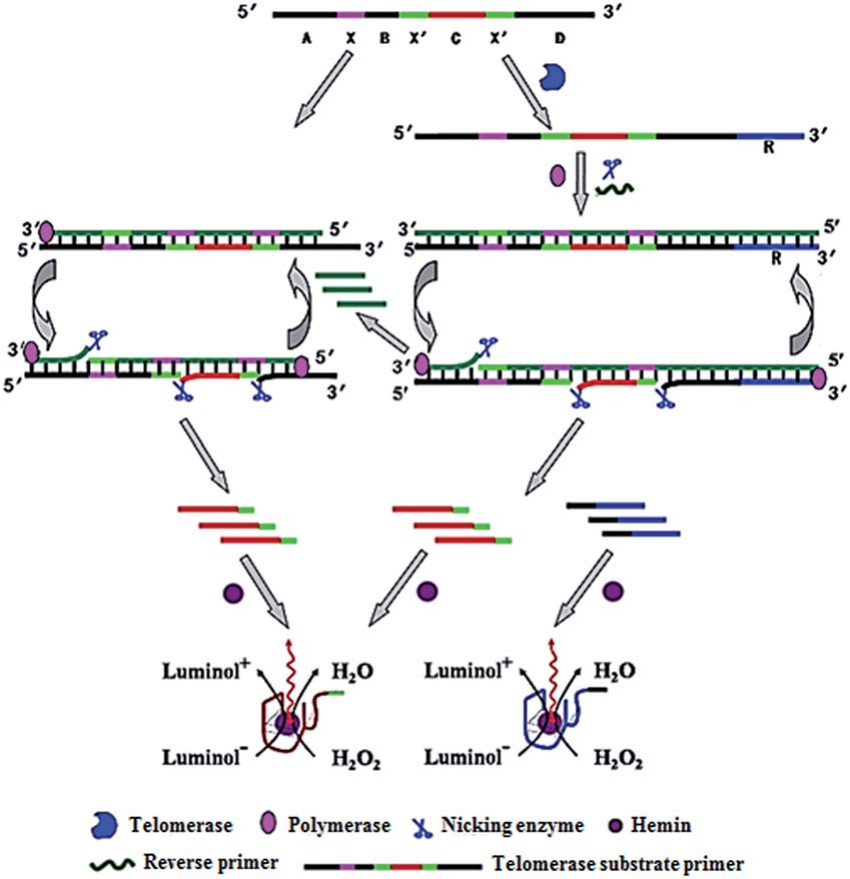
Integration of telomerase-induced two-stage isothermal amplification with chemiluminescent assay for ultrasensitive detection of telomerase activity. Adapted with permission from ref. 48, Copyright 2014 American Chemical Society.

Zhang demonstrated the use of two-stage isothermal amplification-mediated chemiluminescence for the ultrasensitive detection of telomerase activity (Fig. 7).^48^ In the presence of telomerase, the substrate primer is extended, producing telomere repeats of (TTAGGG)_*n*_, which may function as the templates for strand displacement amplification (SDA). With the addition of primer, polymerase and nicking enzyme, multiple catalytic DNAzyme sequences and telomere repeats of (TTAGGG)_*n*_ are generated. The resultant telomere repeats may subsequently function as the primers to trigger an isothermal exponential amplification reaction (EXPAR) and generate numerous catalytic DNAzyme sequences. These catalytic DNAzyme sequences can bind hemin to form G-quadruplex nanostructures which may catalyze the generation of luminol-mediated chemiluminescence signals. Meanwhile, in the absence of telomerase, no amplification reaction is initiated, and no chemiluminescence signal is observed. This assay is highly sensitive, and it can detect the telomerase activity from a single HeLa cell without the involvement of any labeled DNA probes.

## 3 *In vivo* imaging of intracellular telomerase activity

In addition to the above *in vitro* telomerase assays, a series of novel imaging methods for *in vivo* detection of telomerase activity have been developed recently. These imaging methods enable real-time tracking of dynamic telomerase processes in living cells, benefiting the study of its physical role in disease development and drug response.

**Fig. 8:**
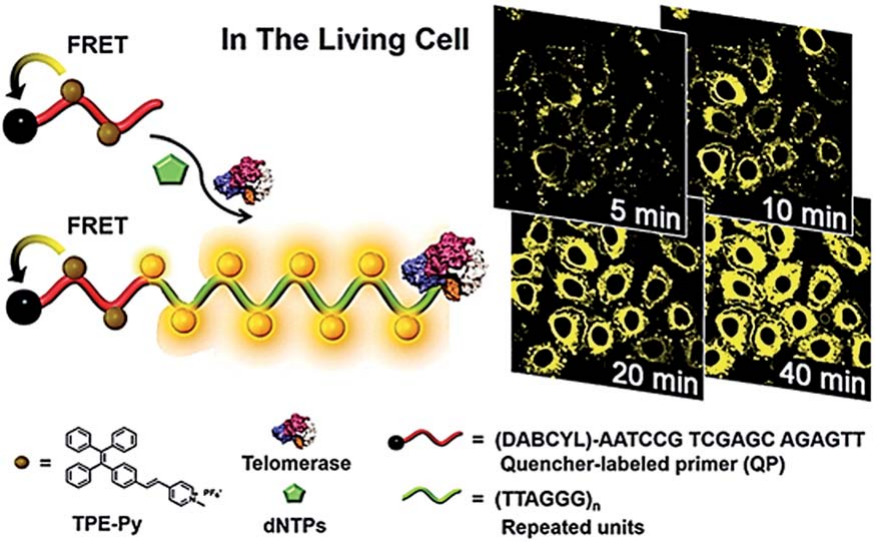
Use of TPE-Py as the AIE dye for *in situ* imaging of telomerase in living cells. Adapted with permission from ref. 49, Copyright 2016 American Chemical Society.

Lou demonstrated the use of positively charged TPE-Py molecules for the imaging of telomerase in living cells (Fig. 8).^49^ The TPE-Py is a kind of aggregation-induced emission (AIE) dye with weak fluorescence in the separation state but intense fluorescence in the aggregation state.^50^ The telomerase substrate is labelled with a quencher. In the absence of telomerase, the fluorescence originating from the binding of TPE-Py to the probe is quenched efficiently as a result of FRET from TPE-Py to the quencher. After being transferred into living cells, the extension of the substrate by telomerase generates a long DNA sequence with repeats of TTAGGG, which can bind TPE-Py molecules to generate a strong fluorescence signal. This assay possesses significant advantages of high stability and superior photostability, holding great potential in clinical diagnosis and telomerase-related drug screening.

**Fig. 9:**
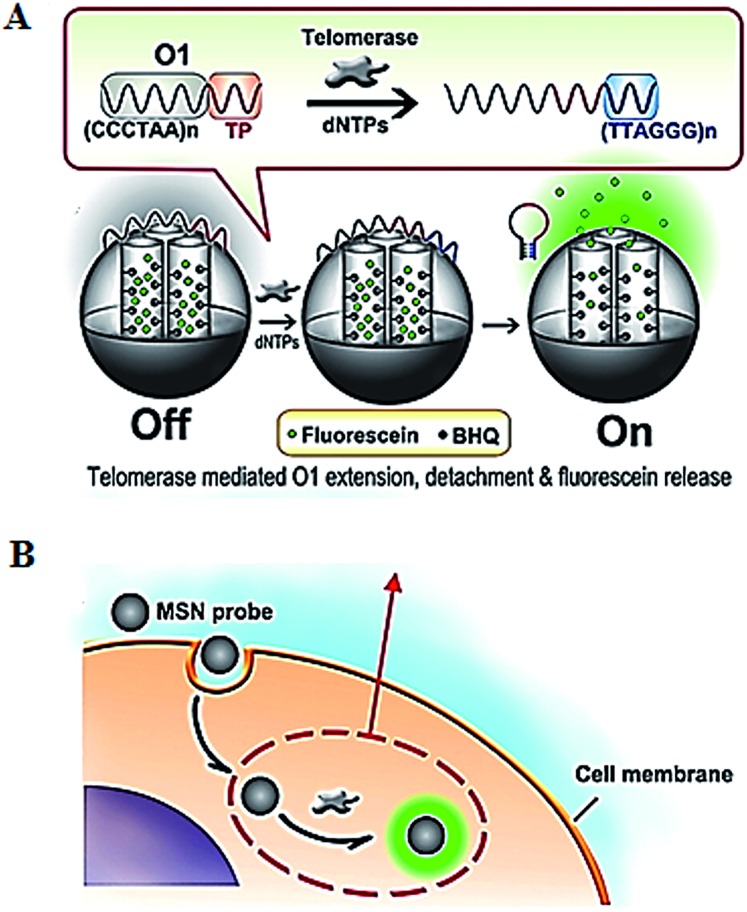
Use of mesoporous silica nanoparticle for real-time imaging of intracellular telomerase activity. Adapted with permission from ref. 52, Copyright 2013 American Chemical Society.

The introduction of novel nanomaterials such as mesoporous silica nanoparticle (MSN) significantly improves the *in vivo* assay performance. The MSN has the distinct characteristics of unique pore structure, biocompatibility and ease of functionalization, and is suitable for intracellular research.^51^ Ju demonstrated the use of a telomerase-responsive mesoporous MSN probe for the fluorescent imaging of intracellular telomerase activity (Fig. 9).^52^ They prepared MSN and the wrapping DNA (O1), respectively. The MSN contains fluorescein in the mesopores and black hole fluorescence quencher (BHQ) on the inner walls of the mesopores. The O1 consists of the telomeric repeats and the telomerase substrate. In the absence of telomerase, the MSN probe is sealed by the O1, and fluorescein is quenched by BHQ. In the presence of telomerase, the extension of substrate by telomerase leads to the removal of the O1 from the MSN surface and consequently the release of fluorescein which can be imaged by confocal microscopy. This assay enables switchable and real-time tracking of telomerase activity in living cells, and it can be used to monitor the change of intracellular telomerase activity in response to drugs.

**Fig. 10:**
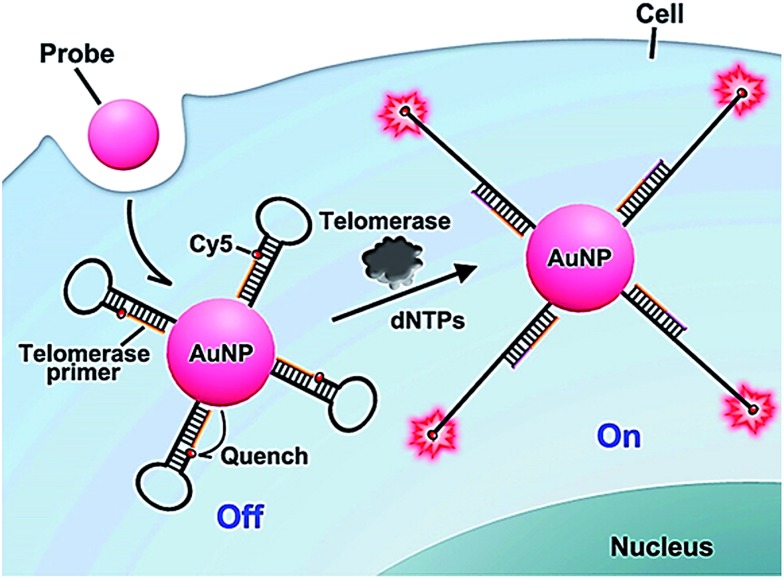
Use of the nicked molecular beacon-functionalized gold nanoparticle for real-time imaging of intracellular telomerase activity. Adapted with permission from ref. 53, Copyright 2014 American Chemical Society.

The above method^52^ involves complicated probe preparation, high background signal and multiple reaction processes. To simplify the procedures, Ju further demonstrated the use of gold nanoparticles and the nicked molecular beacon for one-step imaging of intracellular telomerase activity.^53,54^ They designed a nicked molecular beacon with a nick at the 5′-end stem which separates the beacon into two parts: the telomerase primer sequence and a loop structure (Fig. 10).^53^ The loop structure is labeled with Cy5 at 5′-end and modified by thiol at the 3′-end. The MB is conjugated to an AuNP *via* the thiol-labeled 3′-end, and the Cy5 is quenched by AuNP *via* FRET between Cy5 and AuNP. In the presence of telomerase, the extension of the primer by telomerase leads to the opening of hairpin and consequently the recovery of Cy5 fluorescence as a result of the separation of Cy5 from the AuNP surface. The Cy5 fluorescence lights up the telomerase. This assay exhibits good specificity and high sensitivity, and it may be applied for the discrimination of tumor cells from normal cells. In addition, Ding demonstrated the integration of nucleic acid-based signal amplification with a molecular beacon for a intracellular telomerase assay.^55^

**Fig. 11:**
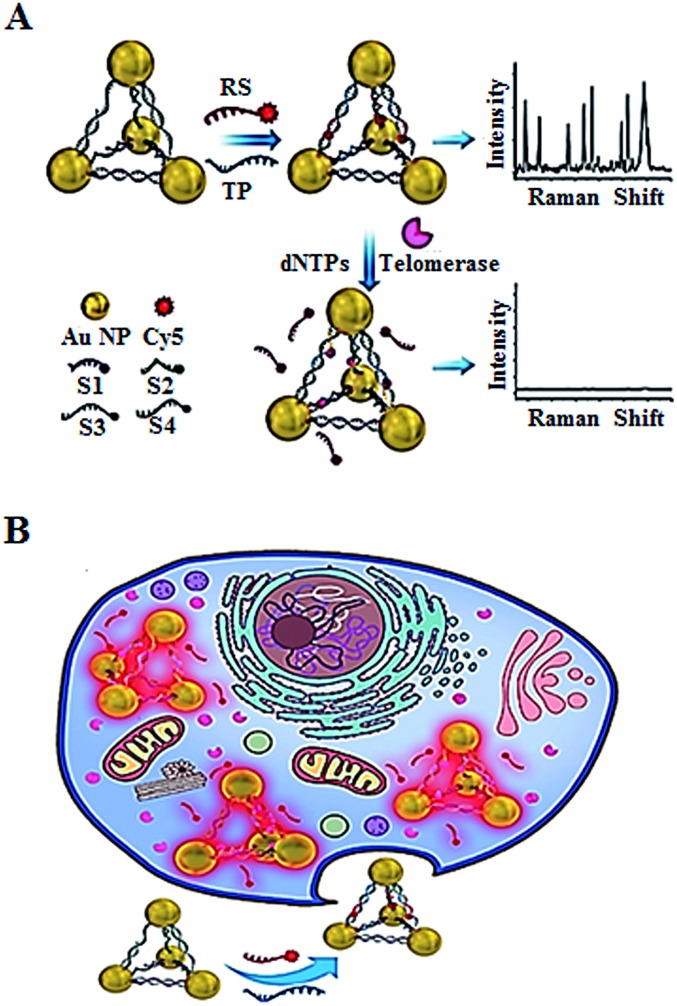
Use of Raman scattering spectroscopy for *in situ* monitoring of telomerase activity in (A) cell extracts and (B) in living cells. Adapted with permission from ref. 56, Copyright 2016 WILEY-VCH Verlag GmbH & Co. KGaA, Weinheim.

In addition to the fluorescent imaging methods,^49,52,53^ Kuang demonstrated the use of *in situ* Raman scattering spectroscopy for the intracellular telomerase assay (Fig. 11).^56^ They designed a Cy5-tagged reporter probe (RS) and four single-stranded DNAs (ssDNAs, S1, S2, S3, and S4) which contain a complementary sequence to the telomerase primer (TP). The TP and RS may hybridize with the specific sequences of the four ssDNAs, leading to the formation of gold nanoparticle pyramids and consequently the generation of a high Raman signal. In the presence of telomerase, the extension of TP by telomerase releases RS from the pyramid scaffold, resulting in a decrease of the Raman signal. This assay may be used for *in situ* monitoring of intracellular telomerase activity in cell extracts with a linear range from 1 × 10^–14^ to 5 × 10^–11^ IU and a detection limit of as low as 6.2 × 10^–15^ IU. Meanwhile, through monitoring the fluorescence signal of the Py-Cy5 nanostructure by confocal microscopy, the intracellular telomerase can also be fluorescently imaged in living cells with a detection limit of 9.6 × 10^–15^ IU. Moreover, this assay shows significant advantages of excellent intracellular stability, biocompatibility and good specificity.

## 4 Summary and outlook

Accurate quantification of telomerase activity plays a crucial role in clinical diagnosis and anti-cancer drug development, and great progress has been made in this area. In this Minireview, we summarize the recent advance in telomerase assays, including both *in vitro* assay and intracellular imaging. The *in vitro* telomerase activity may be monitored by a variety of approaches such as fluorescent,^30,31,33,37^ colorimetric,^38^ electrochemiluminescent,^41^ Raman scattering spectroscopy,^47^ and chemiluminescent assays.^48^ These *in vitro* assays possess the advantages of simplicity, good selectivity and high sensitivity. They cannot, however, be applied for *in vivo*/*in situ* monitoring of intracellular telomerase activity. The *in vivo* imaging of telomerase may provide direct information about its role in cancer progression and its response to drug treatment. With the introduction of novel nanomaterials, the *in vivo* imaging of telomerase in living cells may be achieved.^49,52,53,56^ Both *in vitro* and *in vivo* telomerase assays make a great contribution to biomedical research and clinical diagnostics.

Notably, it still remains a great challenge to develop an ideal telomerase assay that satisfies the demands of rapidity, simplicity to operate, low cost, high sensitivity, good selectivity, and high-throughput at the same time. In addition, the *in vivo* monitoring of telomerase is still in its initial stage, and great efforts should be put into improving its performance in a complex cell environment. With the discovery of novel nanomaterials and the introduction of both new fluorescent labels and efficient labelling strategies, we believe that the development of *in vitro*/*in vivo* telomerase assays may greatly facilitate clinical diagnosis and drug screening in the near future.
